# Outcomes in oncogenic-addicted advanced NSCLC patients with actionable mutations identified by liquid biopsy genomic profiling using a tagged amplicon-based NGS assay

**DOI:** 10.1371/journal.pone.0234302

**Published:** 2020-06-11

**Authors:** Jordi Remon, Aurelie Swalduz, David Planchard, Sandra Ortiz-Cuaran, Laura Mezquita, Ludovic Lacroix, Cecile Jovelet, Etienne Rouleau, Camille Leonce, Frank De Kievit, Clive Morris, Greg Jones, Kelly Mercier, Karen Howarth, Emma Green, Maurice Pérol, Pierre Saintigny, Benjamin Besse

**Affiliations:** 1 Department of Cancer Medicine, Gustave Roussy, Villejuif, France; 2 Centre Leon Berard, Lyon, France; 3 Laboratoire de Recherche Translationnelle, Gustave Roussy, Villejuif, France; 4 Inivata, Granta Park, Cambridge, United Kingdom; 5 Université Paris-Sud, Orsay, France; Universidade do Algarve Departamento de Ciencias Biomedicas e Medicina, PORTUGAL

## Abstract

Circulating tumor DNA (ctDNA)-based molecular profiling is rapidly gaining traction in clinical practice of advanced cancer patients with multi-gene next-generation sequencing (NGS) panels. However, clinical outcomes remain poorly described and deserve further validation with personalized treatment of patients with genomic alterations detected in plasma ctDNA. Here, we describe the outcomes, disease control rate (DCR) at 3 months and progression-free survival (PFS) in oncogenic-addicted advanced NSCLC patients with actionable alterations identified in plasma by ctDNA liquid biopsy assay, InVisionFirst®-Lung. A pooled retrospective analysis was completed of 81 advanced NSCLC patients with all classes of alterations predicting response to current FDA approved drugs: sensitizing common EGFR mutations (78%, n = 63) with T790M (73%, 46/63), ALK / ROS1 gene fusions (17%, n = 14) and BRAF V600E mutations (5%, n = 4). Actionable driver alterations detected in liquid biopsy were confirmed by prior tissue genomic profiling in all patients, and all patients received personalized treatment. Of 82 patients treated with matched targeted therapies, 10% were at first-line, 41% at second-line, and 49% beyond second-line. Acquired T790M at TKI relapse was detected in 73% (46/63) of patients, and all prospective patients (34/46) initiated osimertinib treatment based on ctDNA results. The 3-month DCR was 86% in 81 evaluable patients. The median PFS was of 14.8 months (12.1–22.9m). Baseline ctDNA allelic fraction of genomic driver did not correlate with the response rate of personalized treatment (p = 0.29). ctDNA molecular profiling is an accurate and reliable tool for the detection of clinically relevant molecular alterations in advanced NSCLC patients. Clinical outcomes with targeted therapies endorse the use of liquid biopsy by amplicon-based NGS ctDNA analysis in first line and relapse testing for advanced NSCLC patients.

## Introduction

Since the identification of driver oncogenic alterations in advanced non-small cell lung cancer (NSCLC), tumor genomic profiling is standard of care in daily clinical practice. The wide selections of approved targeted therapies have impressively improved clinical outcomes, specifically tyrosine kinase inhibitors (TKI). Tumor biopsy is the preferred approach for molecular testing, but comprehensive and timely tissue genotyping is challenging as it is invasive. Additionally, inadequate quality tissue is reported for testing in up to one third of cases [1,2]. Recently in the NILE study it was reported that baseline tissue genotyping for all eight guideline-recommended biomarkers in NSCLC was only completed in 18.1% of patients [3]. Similarly, rebiopsy at the time of TKI progression is not always feasible nor informative [4], leading to significant numbers of patients being under-genotyped or non-genotyped for genomic biomarkers recommended by professional guidelines [5]. These clinical guidelines, including an expert committee convened by the International Association for the Study of Lung Cancer (IASLC), advocate comprehensive genomic profiling (CGP) using next-generation sequencing (NGS) technology by circulating tumor DNA (ctDNA) testing at baseline or at the time of progression to personalized treatment when tissue biopsy is infeasible or inadequate for molecular analysis [6]. ctDNA testing analyses patient blood samples for somatic sensitizing and resistance alterations and fusions in the fragments of tumor DNA. This providing a non-invasive, simple blood test as an alternative to tissue biopsy. Contrary to tissue acquisition feasibility, different cohorts have reported that ctDNA testing result in guideline complete genotyping in up to 95% of NSCLC patients [3,7,8]. Several reports have further demonstrated analytical and clinical validation of ctDNA liquid biopsy in NSCLC and other tumor types, however, discordance between tissue- and plasma-based NGS sequencing tests remains evident [9,10]. The differentiation of performance between liquid biopsy assays highlights the importance of the choice of tests being used in clinical practice that require robust analytical and prospective clinical validation data [11].

Clinical outcomes in patients with positive actionable alterations detected using liquid biopsies is still scarce [8,12,13]. More data is needed to endorse the clinical utility and validity of this technique in daily clinical practice. Depth of response rate may provide an additional outcome measure for evaluating treatment activity in oncogenic-addicted NSCLC patients treated with TKI [14]. Likewise, time to treatment failure has been cited by the US Food and Drug Administration (FDA) as a surrogate endpoint for clinical impact of targeted therapy [15]. The amount of DNA being shed by the tumor is measured by the variant allele fraction (AF) in plasma, which correlates with the location and volume of disease as well as the amount of non-tumoral DNA circulating at the time of the blood draw. The correlation between plasma AF (%) and the response rate on targeted therapies as assessed by Response Evaluation Criteria in Solid Tumors (RECIST) remains inconclusive, with some previous data reporting a lack of correlation [8,12]. Herein, we describe clinical outcomes with targeted therapies in a daily clinical practice population of advanced NSCLC patients with actionable alterations identified by an amplicon-based NGS assay, the InVisionFirst^®^-Lung, with the aim to assess the feasibility and clinical relevance of liquid biopsy testing in this population.

## Materials and methods

This pooled retrospective analysis combined advanced NSCLC patients from 3 studies conducted in the United States and France: Liquid Biopsies in Patients Presenting Non-Small Cell Lung Cancer (LIBIL) study (NCT02511288); Liquid Biopsy (NCT02666612), and Reveal study (NCT02906852). Patients were included in the analysis if they met the following criteria: 1) stage IIIB/IV NSCLC, with a positive InVisionFirst^®^-Lung ctDNA liquid biopsy for an actionable alteration (common sensitizing and resistant *EGFR* mutations, *BRAF* V600E mutation or *ALK/ROS1* fusion) with or without concurrent tissue biopsy, and 2) treated with an appropriate targeted therapy, namely FDA-approved TKIs according to the mutation identified. Patients were prospectively enrolled between 2015 and 2018 and eligible patients were consented by the Institutional ethic committee that approved the studies. This retrospective pooled analysis did not require specific approval, as data was de-identified.

**Fig 1:**
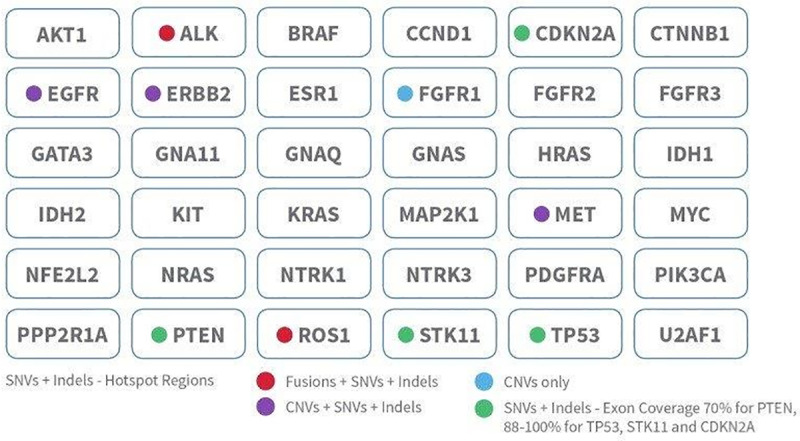
The InVisionFirst®-Lung assay identifies SNVs, indels, CNVs and gene fusions with whole gene and gene hotspots, using an amplicon-based NGS technology.

InVisionFirst®-Lung is a tagged amplicon-based NGS Laboratory Developed Test (LDT) (Inivata, Research Triangle Park, NC, US and Cambridge, UK) that is used to identify somatic genomic alterations in all four variant types: single nucleotide variants (SNVs), Insertions/Deletions (InDels), Copy Number Variants (CNVs) and Structural Variants (SVs)/Fusions, within a focused 36 gene panel (Fig 1) according to methods previously described [16].

The objectives of the study were to evaluate the disease control rate (DCR) at 3 months in patients treated with matched targeted therapies according to InVisionFirst®-Lung results, progression free survival (PFS) on TKI treatment and assess the correlation between baseline AF in ctDNA and clinical outcomes. A pooled-analysis was performed: patients treated with matched targeted therapies according to InVisionFirst®-Lung results and evaluable for DCR at 3 months were collated for clinical outcomes analysis. Survival analyses were performed to describe overall PFS and stratified by treatment group, and baseline variant AF (%). For the Kaplan-Meier survival analysis, the AF% groups were chosen by the previous observation where 25% of patients with acquired *T790M* mutations were found at an AF% of <0.5% [12]. Correlation between AF and response rate (RR) according to RECIST 1.1 criteria by investigator was assessed in the whole population as well as in *T790M* positive subgroup [17]. Patients on therapy at their last documented assessment were censored in this analysis. Mann-Whitney ranked sum test was performed on group comparisons. Statistics were generated by MedCalc Statistical Software version 18.11.3 [18].

## Results

The study included 81 patients (69% female, 96% stage IV, 58% never smokers, 63 *EGFR* (78%, with 46 T790M (73%), 14 *ALK/ROS1* fusion (17%), 4 *BRAF* V600E (5%) (S1 Table). Patient characteristics are described in Table 1. Driver mutations detected in liquid biopsy were confirmed by prior tissue genomic profiling in all patients, and all received personalized treatment matching these genomic alterations with 10%, 41% and 49% receiving treatment at first-line, second-line or beyond, respectively. In *EGFR* mutant population, acquired T790M was identified by ctDNA assay in 54% (34/63) of patients, all without concurrent tissue genotyping and received matched therapy (osimertinib) solely according to liquid biopsy results.

**Table 1:** Patient characteristics.

	n (%)
**Age**	64
**Sex–Female**	56 (69.1)
**Cancer stage–IV**	78 (96.3)
**Histopathology**	
**Adenocarcinoma**	76 (93.8)
**Smoking History**	
**Never**	47 (58.0)
**Smoker**	31 (38.3)
**Prior therapy lines**	
**Median (Range)**	1 (0–11)

**Fig 2:**
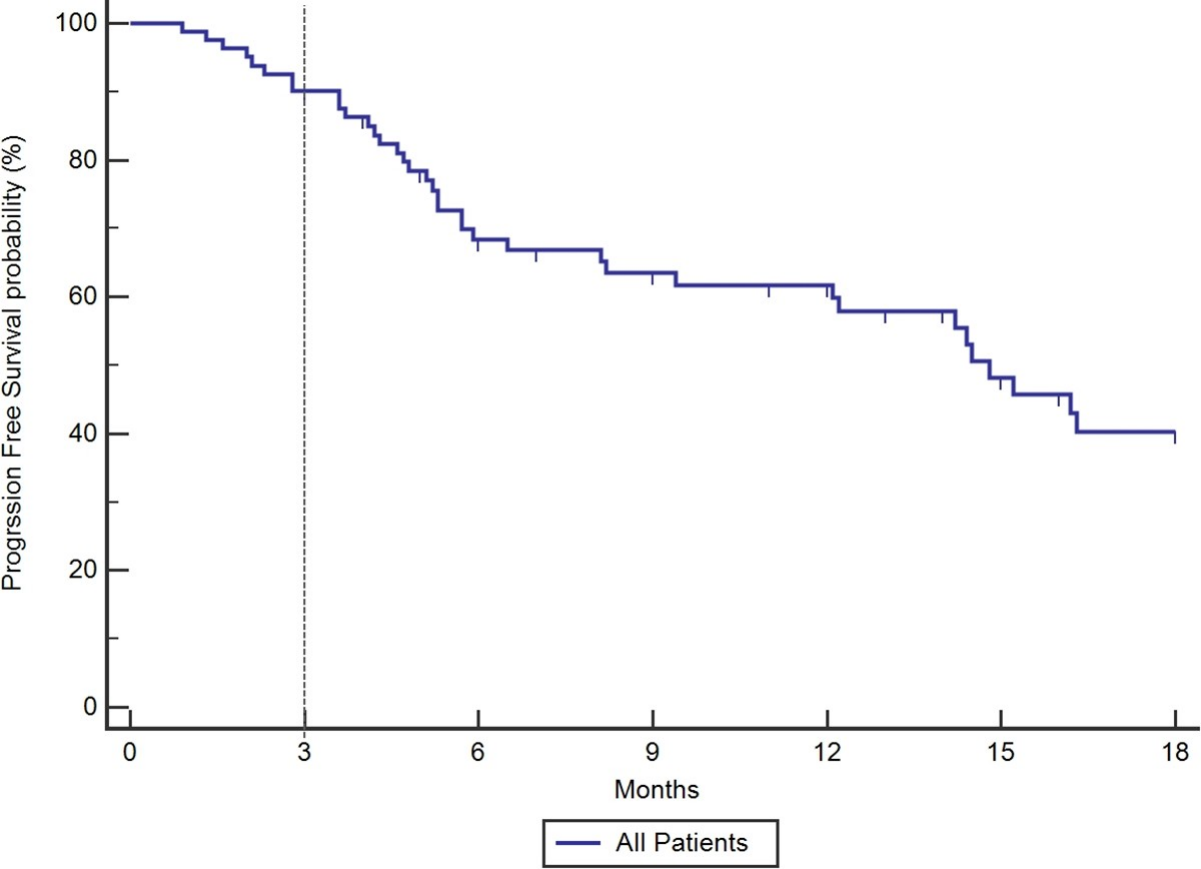
Kaplan Meier curve for progression-free survival (median, 14.8m) in overall cohort of patients treated with targeted therapies matched by genomic profiling, irrespective of therapy line. 90% of patients were progression-free at 3 months on therapy.

**Fig 3:**
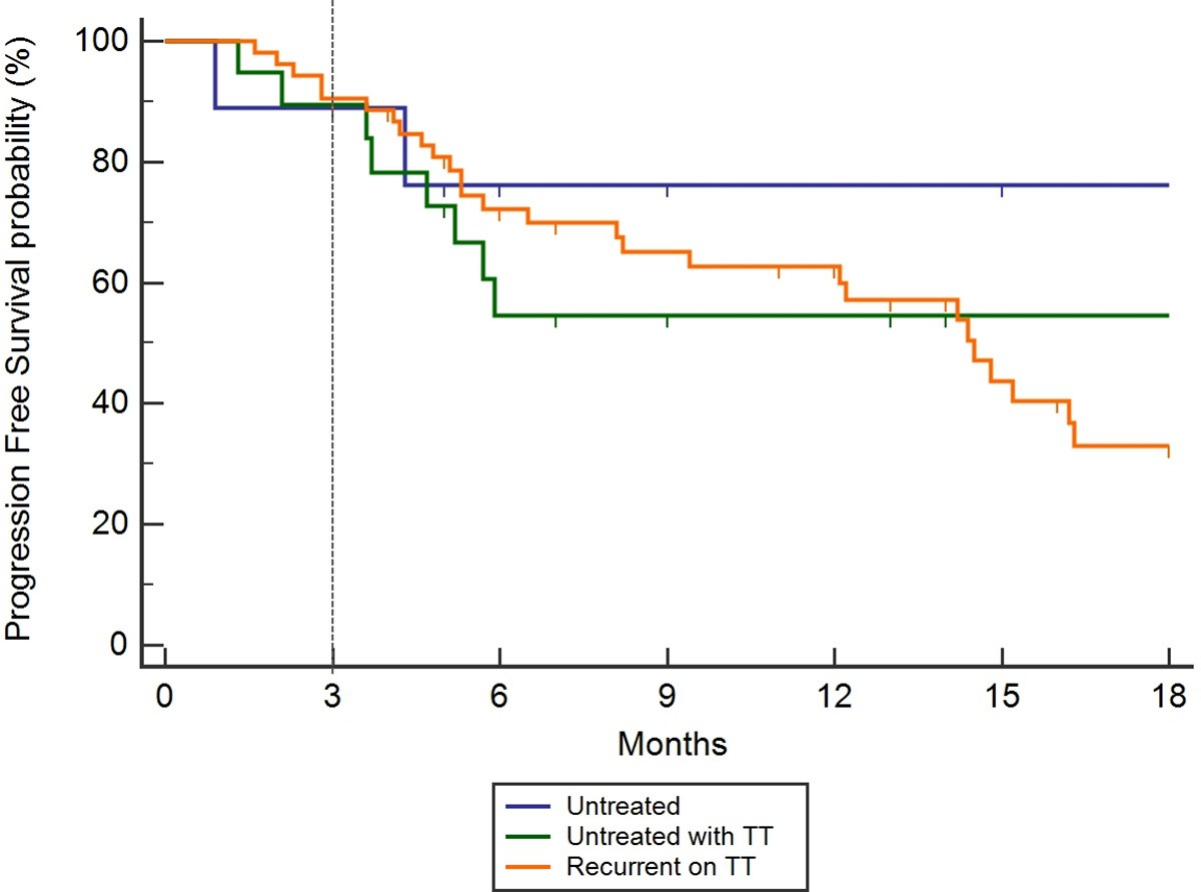
Kaplan Meier curve with stratification of groups, demonstrating the progression-free survival (PFS) for patients on an appropriate targeted therapeutic (TT) agent as determined by the identification of an actionable mutation. No significant difference was identified between patients who were untreated, TKI-naïve or recurrent to prior TKI therapy (p = 0.8552).

The response rate at 3 months in the whole population was 62%. The 3-month DCR on targeted therapies was 86% (70/81) in evaluable patients. The DCR at 3 months was 85% among TKI-naïve (N = 27) patients. Similarly, the DCR at 3 months was 87% for TKI-pretreated patients with progression who received a new TKI (N = 54). The PFS rate at 3 and 6 months was of 90% and 68%, respectively, with a median PFS of 14.8 months (12.1–22.9 months) (Fig 2). There was no significant difference in PFS between patients who were untreated, TKI-naïve or recurrent to prior TKI therapy (p = 0.8552) (Fig 3). The DCR at 3 months according to the genomic profile was 87%, 100%, and 50% for *EGFR* mutant, *ALK /ROS1* fusion, and *BRAF* V600E mutant, respectively (Table 2).

**Table 2:** Disease control rate at 3 months according to InVisionFirst results in 82 patients receiving FDA-approved targeted therapies.

Prior therapy for advanced disease	Genomic alteration	N	Number still on targeted therapy at 3 months	% still on targeted therapy at 3 months
**Untreated for advanced disease**	all	9	7	78%
	*EGFR* Mutation	6	5	83%
	*BRAF* V600 mutation	2	1	50%
	*ALK/ROS1* Fusion	1	1	100%
**Prior cytotoxic chemotherapy for advanced disease but no targeted therapy**	all	18	16	89%
	*EGFR* Mutation	9	8	89%
	*BRAF* V600 mutation	2	1	50%
	*ALK/ROS1* Fusion	7	7	100%
**Prior therapy with targeted therapy**	all	54	47	87.0%
	*EGFR* Mutation	48	41	85.4%
	*ALK/ROS1* Fusion	6	6	100%
**Overall**		81	70	86.4%

**Fig 4:**
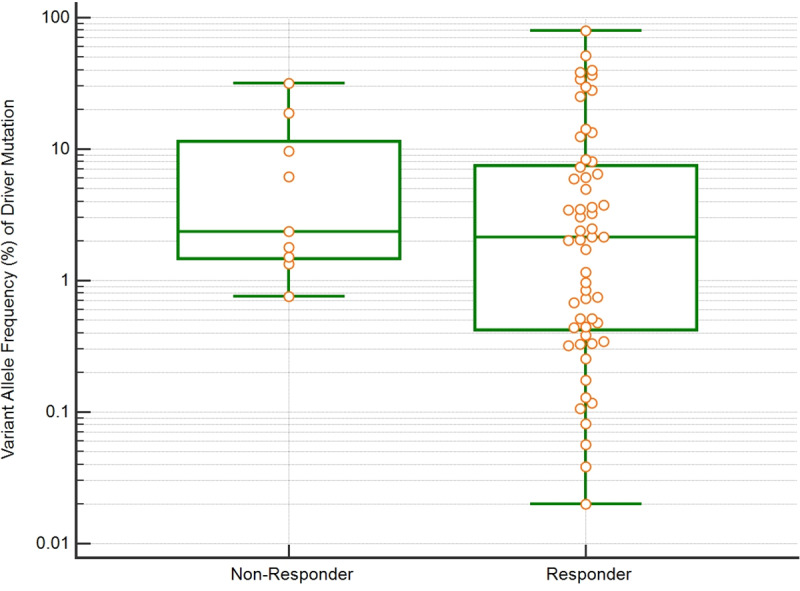
Whisker box-plot demonstrating baseline driver variant allele fraction (AF%) for each patient stratified by response or no response to therapy: In the overall population, baseline VAF was not associated with disease control at 3 months (p = 0.2911).

**Fig 5:**
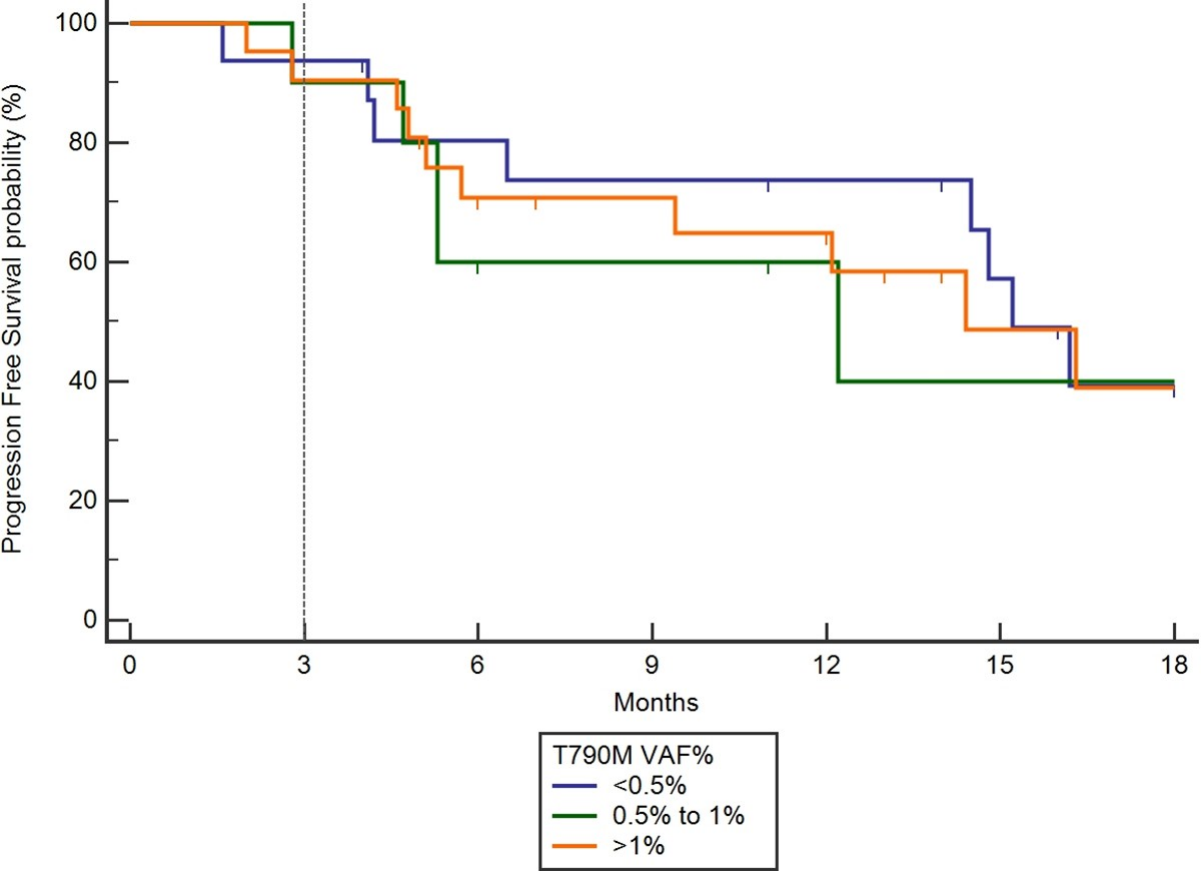
Kaplan Meier PFS curve in cohorts segregated by the magnitude of the measured T790M at disease recurrence. PFS of patients with low variant allele fraction (<0.5%) was compared to higher levels (0.5% to 1.0% or >1%). Comparison of groups by Logrank test, CHI squared 0.6021, p = 0.7401 showed no difference in PFS benefit at higher allele fractions vs low allele fractions.

In the overall population, the baseline AF did not correlate with DCR at 3 months (p = 0.2911, Fig 4). The baseline AF of acquired T790M showed neither correlation with RR nor to PFS before starting osimertinib. The PFS rate at 3 months of patients with low levels of T790M (AF <0.5%) was compared to those with AF at higher levels (0.5% to 1% or >1%) (Fig 5), and no advantage in PFS was observed with higher levels of T790M versus those patients with AF at less than 0.5%. The median PFS was of 15.2 months, 12.2months and 14.4 months for AF of 0.5%, for AF 0.5–1% and for AF ≥ 1%, respectively, p = 0.9656.

## Discussion

In this multicenter ctDNA pooled analysis utilizing a tagged amplicon-based NGS assay, the InVisionFirst®-Lung assay, we report that personalized treatment in oncogenic-addicted advanced NSCLC patients according to liquid biopsy genomic profile achieved a 3-months DCR of 86%. This corresponds with previous data reported in 42 patients treated with targeted therapies based on liquid biopsy results by the Guardant Health 360 assay [8]. Indeed, in our cohort a median PFS on TKI was of 15 months. This is despite almost all patients having had initiated personalized treatment in second line or beyond, suggesting that personalised treatment beyond progression may impact in patients’ clinical outcomes. Our cohort was enriched with *EGFR*-mutant NSCLC patients, and no patient was treated with upfront osimertinib. Similarly, any *ALK*-positive NSCLC patient was treated with upfront next-generation ALK TKI. The efficacy of next-generation EGFR or ALK TKI in TKI-refractory patients is similar to data reported with upfront erlotinib or crizotinib, respectively [19–22]. All these data could explain the lack of difference in DCR and PFS in TKI-naïve and pre-treated patients in our cohort. However, we cannot rule out that may exist a difference in the case that patients enrolled had been treated with next-generation TKI in first-line setting or in enlarged cohort.

The data in our cohort mirrors the DCR on targeted therapy reported in tissue genotyping NSCLC patients, supporting the clinical utility of liquid biopsy as a reliable tool for making treatment decisions without a negative impact in patients’ outcomes. Although tissue biopsy remains the standard of care (SoC), in the setting of inadequate tissue, these results show that liquid biopsy can be an adequate surrogate for tissue comprehensive genomic profiling as part of routine clinical care for patients with metastatic NSCLC. Currently, tumor genotyping is critical as personalized treatment for *EGFR*, *ALK*, *ROS1*, *BRAF* in the first-line setting. It also is SoC at the time of progression on TKI for *EGFR* and *ALK* NSCLC patients [23]. Despite this relevance, under-genotyping for all guideline recommended biomarkers, continues to challenge the treatment of advanced NSCLC patients. Recently, two different cohorts of newly diagnosed NSCLC patients have reported that only 8% to 18% of patients had complete tissue genotyping for all guideline-recommended biomarkers [3,24], with almost 20% not tested for *EGFR* mutations or *ALK* fusions, 40% untested for ROS1 fusions, and more than 75% untested for the *BRAF* V600E mutation [3,24]. In contrast, different cohorts assessing the utility and feasibility of cfDNA testing for genomic profile in advanced NSCLC patients resulted in guideline compliant complete genotyping in almost 95% of patients with a concordance rate between tissue and liquid biopsy of up to 90% [3,7,8]. Additionally, there were shorter turnaround time for genomic profiling using ctDNA compared with tissue genomic profiling (10 days versus 15 days) [3]. However, due to the lack of detection of mutations in ctDNA in up to 30% of patients in previous studies, liquid biopsy is not recommended as a replacement for tissue [25]. In two prospective studies using InVisionFirst®-Lung in untreated advanced NSCLC patients one or more cancer-related genomic alterations were reported in 70% and 77% of patients, with a clinical sensitivity of 73.9% and specificity of 99.8% [7,26]. This is of relevance as additional targets on this panel remain under clinical investigation, suggesting that the number of personalized treatment options will increase overtime. Tissue profiling also presents a challenge due to inter and intra-tumor heterogeneity, as biopsies taken from one region of tumor may not represent the genomic profile of the entire tumor or of the metastatic tumor sites distal to the primary tumor [27]. This explains that liquid biopsy may rescue almost 20% of wild-type tissue genotyping patients who can get benefit of personalised treatment [3,7,8]. Therefore, liquid biopsy is recommended as a complementary alternative to tissue biopsy for genomic profiling.

As the sensitivity of ctDNA assays improves, the question arises regarding whether therapeutic targeting of a low AF mutation will yield clinical benefit. In the studied cohort, the AF did not correlate with the response rate on TKI therapy. Likewise, the RR and PFS on osimertinib in acquired T790M mutant NSCLC patients were independent of baseline AF according to three pre-defined subgroups (<0.5%, 0-5-1% and ≥ 1%). Previous studies have reported a lack of concordance between AF and RR on osimertinib [8,12,28] or with the whole population of oncogenic addicted tumors receiving other targeted therapies [8]. This lack of concordance could be correlated with tumor characteristics such as tumor volume and number of metastatic sites, which was not collected in this cohort.

One key limitation to this study is that, while cfDNA testing utilized a single platform, tissue genomic assessment was not standardized. We cannot rule out some percentage of false-negative results in the tissue genotyping. Another limitation is that while these studies prospectively enrolled patients, two of the studies were observational with a limited number of patients included. Finally, the outcome parameters (RR, PFS) were assessed by the investigator and not centralized. The exception is that the osimertinib cohort (34 patients out of whole population) RR assessment was centralized. This could lead to an overestimation of the PFS of the whole population based on the clinical option of treatment beyond progression [29].

We provide evidence that oncogenic drivers detected in ctDNA result in treatment response in patients that is comparable to that seen in tissue irrespective of the variant allele fraction. This suggesting that a robustly validated NGS-based test with cfDNA can match or even improve upon SoC tissue methods as the percentage of complete genomic profile is higher, endorsing both its feasibility, and providing clinically relevant alternative to tissue.

## Conclusions

ctDNA molecular profiling is an accurate and reliable tool for the detection of clinically relevant molecular alterations in advanced NSCLC patients. Clinical outcomes with targeted therapies endorse the use of liquid biopsy by amplicon-based NGS ctDNA analysis in first line and relapse testing for advanced NSCLC patients.

## Supporting information

S1 Table (XLSX)
